# Explaining the effects of a point-of-purchase nutrition-information intervention in university canteens: a structural equation modelling analysis

**DOI:** 10.1186/1479-5868-9-111

**Published:** 2012-09-11

**Authors:** Christine Hoefkens, Zuzanna Pieniak, John Van Camp, Wim Verbeke

**Affiliations:** 1 Department of Food Safety and Food Quality, Ghent University, Faculty of Bioscience Engineering, Coupure Links 653 B-9000, Ghent, Belgium; 2 Department of Agricultural Economics, Ghent University, Faculty of Bioscience Engineering, Coupure Links 653 B-9000, Ghent, Belgium

**Keywords:** Point-of-purchase information, University canteen meals, Young adults, Information processing, Motivation, Nutrition knowledge, Moderated mediation model, Structural equation modelling

## Abstract

**Background:**

The importance of canteen meals in the diet of many university students makes the provision of simple point-of-purchase (POP) nutrition information in university canteens a potentially effective way to promote healthier diets in an important group of young adults. However, modifications to environments such as the posting of POP nutrition information in canteens may not cause an immediate change in meal choices and nutrient intakes. The present study aimed at understanding the process by which the POP nutrition information achieved its effects on the meal choice and energy intake, and whether the information was more effective in changing the meal choice of subgroups of university canteen customers.

**Methods:**

The POP nutrition-information intervention used a one-group pretest-posttest design. A sample of 224 customers of two university canteens completed the baseline and 6-months follow-up surveys. A multi-group structural equation modelling analysis was used to test mediation effects of individual difference variables (liking, understanding and use of the information, subjective knowledge and attitude) on the energy intake from canteen meals, moderated by the objective nutrition knowledge and motivation to change diet.

**Results:**

Significant relations were identified between liking of the information and its use on one hand and a positive effect in attitude towards healthy canteen meals on the other hand. Motivation to change diet and sufficient objective nutrition knowledge were required to maintain a recommended energy intake from canteen meals or to lead to a decrease in energy intake. Participants with greater objective nutrition knowledge had a greater understanding of the POP nutrition information which also resulted in a more effective use of the information.

**Conclusions:**

The results suggest that nutrition-information interventions may be more effective when using nutrition information that is generally liked by the target population in combination with an educational intervention to increase objective nutrition knowledge.

**Trial registration:**

NCT01249508

## Background

Young adults often establish unfavourable dietary habits when leaving the parental home to enter university, i.e. consuming a diet of limited variety, high snacking, consuming more high-fat foods (including fast foods), more soft drinks, and less fruit and vegetables [1-3]. Such habits may have a long-lasting impact on their own health or the health of their future families [3,4]. Therefore, it is important to promote maintenance of adequate nutritional habits learned at home or to improve current eating habits.

For many university students canteen meals constitute an important part of the diet [5]. Because canteen customers might not be aware of the nutritional quality of their meal choices [6], which are often too rich in energy, fat and sodium, and contain insufficient amounts of fruit and vegetables [7,8], dietary guidance through simplified point-of-purchase (POP) nutrition information on menu choices in canteens could be a strategically important approach to promote healthy dietary choices.

Evaluation of the overall effect of a POP nutrition-information intervention in two canteens of Ghent University showed that nutrition information by using a star-rating system as signage did not effectively change meal choices and nutrient intakes [9]. Modifications to the environment such as posting nutrition information in university canteens might not cause an immediate dietary change [10]. Consumer behaviour and information processing models posit that communication and information efforts, if being attended to and properly processed, move individuals through a sequence of hierarchical stages, often referred to as a “hierarchy of effects” [11,12]. This concept indicates the different mental stages that consumers go through after being exposed to information and when responding to information and making buying decisions. While it is generally accepted that a structure includes a cognitive response (learning, knowing), an affective response relating to attitude formation (thinking, feeling) and (ultimately) a behavioural response (intending, doing), the sequence and separation of these hierarchical steps depend on person-related, product-related and situational factors. In this study, the classical sequence from knowledge to behaviour was assumed [13,14]. Furthermore, direct paths from each step of the hierarchy to the behavioural stage were investigated.

In addition, the effect of nutrition information on dietary behaviour may differ between individuals [15,16]. To examine causal pathways of information effects in subgroups of canteen customers, moderated mediation models are especially valuable. In these statistical models, a third variable mediates the effect of an independent variable on the dependent variable, and this mediated or indirect effect depends on the level of a moderator (i.e. conditional indirect effect) [17]. Despite the acknowledged importance of investigating mediation and moderation effects of interventions on dietary behaviour, only a few studies have done so and none of them have evaluated a nutrition-information intervention in a canteen environment [18,19].

**Figure 1:**
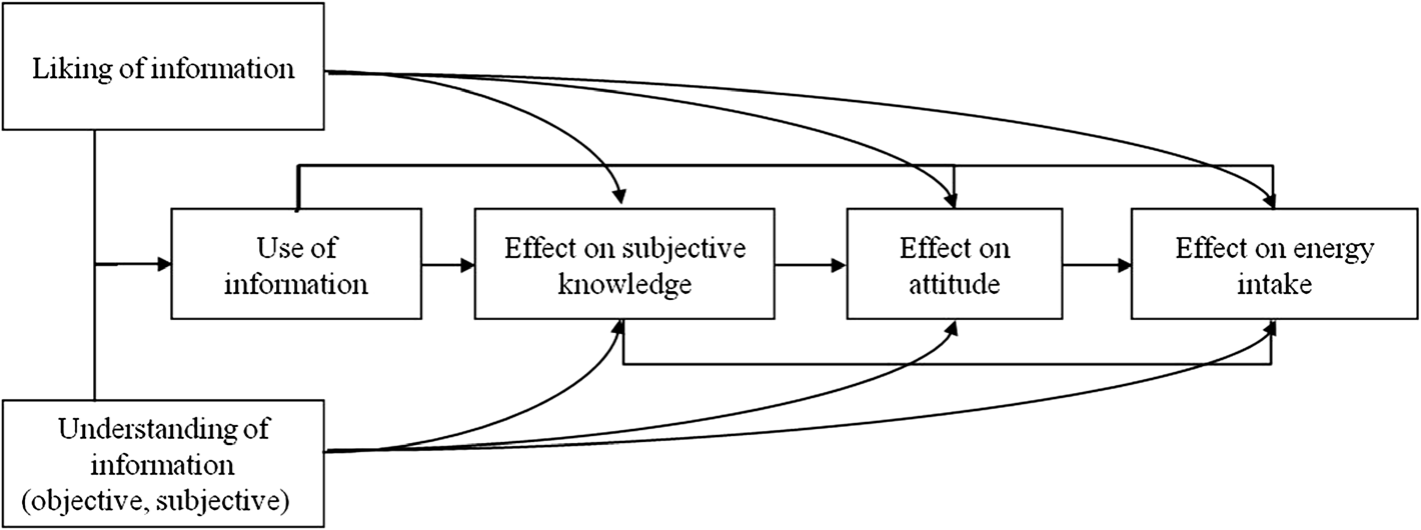
Hypothetical model of the process by which the nutrition information achieves its effects on the meal choice and energy intake.

The objective of the study was to explain the ineffectiveness of our nutrition-information intervention in university canteens [9]. A moderated mediation model was estimated to examine, first, the process by which the POP nutrition information achieved its effects on the meal choice and energy intake, and second, whether the information was more effective in changing the meal choice of subgroups of university canteen customers. From consumer behaviour models, our first hypothesis was that individuals who understand and like the POP nutrition information, will be more likely to use the information, will increase their subjective knowledge about how to evaluate the healthiness of a food, leading to a more positive attitude towards healthy canteen meals and ultimately to a healthier meal choice (Figure 1). A second hypothesis was that the POP nutrition information would be most effective among more motivated and more knowledgeable individuals [16]. Because the information was designed to facilitate the identification of healthier meal choices, also less knowledgeable consumers with a high motivation to change their diet were hypothesized to be positively influenced by the intervention.

## Methods

### Study design and participants

The nutrition-information intervention that forms the starting point of the present study used a one-group pretest-posttest design. A convenience sample of 224 students (165 females and 59 males) between the ages of 17 and 35 years (Mean 21 years, SD 3), who were regular customers of two canteens of Ghent university (Belgium), enrolled in the intervention (59% of the baseline sample) and completed three-day food records and self-administered structured questionnaires at baseline (October and November 2008) and follow-up (April and May 2009).

Customers of the canteens of Ghent University compose their meal by choosing one protein component, one sauce component, one vegetable component and a carbohydrate source. As such, about 180 meal combinations are possibly chosen and consumed daily. Because of display place constraints and the risk of information overload, each day a selection of 12 meals (ie, the three best meal options for each of the four protein components) was communicated. These 12 best meal combinations were selected based on the compliance of a meal’s content of energy, saturated fat, sodium, and the size of the vegetable portion with the respective meal recommendations (for energy: <500 kcal, saturated fat: <13% of energy, sodium: <2.2 mg/kcal, and vegetable: >150 g [20-22]).

The nutrition information on the best meal combinations, which was first posted in March 2009, consisted of a star rating ranging from zero to three stars and a descriptor for nutrients or food group that did not comply with recommendations. If a meal complied with a recommendation, it received a score of 1. The maximum score was 4, which would mean that the meal complied with the meal recommendation for energy, saturated fat, sodium and the amount of vegetables. These scores were translated into stars, whereby scores 2, 3, and 4 received, respectively, 1, 2, and 3 stars. Participants were not informed about the posting of nutrition information in the university canteens but only about the study purpose of assessing eating habits. They were explained that measurements at two points in time were necessary to have a proper representation of consumption patterns. Other measures taken to reduce possible bias from experimenter demand effects, were the posting of the labels in the canteens before opening hours and the formatting of supportive material according to the house style of all communications by the canteen administration. As such, the posted nutrition information and other materials did not contain any link to the study or researchers. A more detailed description of the nutrition-information intervention, the sample characteristics and results of the overall efficacy of the intervention are provided in Hoefkens et al. [9]. The study protocol was granted ethics approval by the Belgian Ethics Committee of the Ghent University Hospital (ethics approval number EC/2008/482) and registered on ClinicalTrials.gov (Id number NCT01249508).

### Measures

#### Behavioural outcome

The primary outcome variable of the intervention was the number of chosen meals that complied with all recommendations (i.e. three-star meals) [9]. The present study used the energy intake from the canteen meal as a proxy for the star rating of the reported meal choice. The energy intake from the canteen meal at both baseline and follow-up were calculated as the average of the three days collected through self-administered food records together with the energy values and standardized portion sizes of the meal components provided by the caterer. Based on the difference in energy intake from the canteen meal between follow-up and baseline, participants were categorized into (1) increasing energy intake (i.e. increase of more than one standard deviation with SD = 147 kcal), (2) maintaining high energy intake, (3) maintaining moderate energy intake, (4) maintaining low or recommended energy intake, and (5) decreasing energy intake (i.e. decrease of more than 147 kcal). The mean (SD) and the distribution of the outcome variable are presented in Table 1.

**Table 1:** Descriptive characteristics (mean ± SD, frequency (%)) for the total sample and subgroups

			**Total sample (n = 220)**^**a**^	**People with low knowledge and high intention (n = 70)**	**People with high knowledge and high intention (n = 44)**
Mediator	**Effect on energy intake**	Mean ± SD	1.10 ± 147	−27.88 ± 132	−30.66 ± 126
	Increase in energy intake	Frequency (%)	5	9	5
	Maintenance of high energy intake		17	10	18
	Maintenance of moderate energy intake		55	57	59
	Maintenance of low or recommended energy intake		16	16	11
	Decrease in energy intake		6	9	7
	**Effect on attitude**	Mean ± SD	−0.69 ± 1.34	−0.61 ± 1.15	−0.64 ± 1.35
	Negative change in attitude	Frequency (%)	10	7	2
	Maintenance of low attitude		23	19	25
	Maintenance of moderate attitude		34	40	32
	Maintenance of high attitude		5	4	7
	Positive change in attitude		27	30	34
	**Effect on subjective knowledge**	Mean ± SD	−0.05 ± 0.94	−0.12 ± 0.99	−0.07 ± 0.71
	Negative change in knowledge	Frequency (%)	10	11	11
	Maintenance of low knowledge		11	13	9
	Maintenance of moderate knowledge		23	30	14
	Maintenance of high knowledge		8	6	5
	Positive change in knowledge		49	40	61
	**Use of information**^**b**^	Mean ± SD	2.90 ± 1.51	3.03 ± 1.47	3.21 ± 1.52
	Never	Frequency (%)	27	24	23
	Rarely		20	16	14
	Occasionally		15	17	14
	Sometimes		24	27	30
	Regularly		10	13	16
	Often		4	1	5
	Always		1	1	0
	**Subjective understanding of information**^**b**^	Mean ± SD	4.54 ± 1.25	4.54 ± 1.23	4.45 ± 1.17
	Totally disagree	Frequency (%)	2	1	0
	Disagree		5	3	5
	Rather disagree		11	13	16
	Neither agree, nor disagree		30	33	34
	Rather agree		26	27	25
	Agree		22	17	18
	Totally agree		4	6	2
	**Objective understanding of information**	Mean ± SD	10.19 ± 2.25	9.63 ± 2.29	10.32 ± 1.91
	**Liking of information**^**b**^	Mean ± SD	4.35 ± 1.06	4.43 ± 1.02	4.56 ± 1.00
	Not like at all	Frequency (%)	2	3	0
	Moderately dislike		3	1	5
	Slightly dislike		12	9	5
	Neutral		34	34	34
	Slightly like		39	41	39
	Moderately like		11	11	18
	Like very much		0	0	0
Moderator	**Objective nutrition knowledge**	Mean ± SD	9.10 ± 1.53	7.03 ± 1.65	11.92 ± 1.76
	**Intention to change diet**^**b**^	Mean ± SD	4.65 ± 1.16	5.53 ± 0.63	5.44 ± 0.58
	Very unlikely	Frequency (%)	2	0	0
	Unlikely		3	0	0
	Rather unlikely		9	0	0
	Neutral		29	0	0
	Rather likely		35	54	59
	Likely		18	37	34
	Very likely		4	9	7

^a^Four individuals were removed from the sample because of incomplete information, leaving a final sample of 220 valid cases.

^b^ Distributional labels for the response categories “2”, “3”, “5”,”6” were not present on the scales at data collection, but are included in the table for clarity of presentation.

#### Theory-based mediators

The theoretical framework of the nutrition-information intervention was based on a combination of the model of consumer information processing proposed by Grunert and Wills [23] and the Hierarchy-of-effects (HOE) model [11].

The hypothesized mediators related to information processing were liking of the information (3 items; Cronbach’s alpha = 0.81) [24,25], subjective understanding (3 items; Cronbach’s alpha = 0.80) [26], objective understanding (aggregated score on 14 items; see further) and self-reported use of the information (5 items; Cronbach’s alpha = 0.93) [27] (Table 1). Liking was measured on a 7-point interval scale from “totally not” to “very much” using the items:”I like the information”, “The information is attractive to me”, “The information is interesting to me”. Subjective understanding of the information was measured on a 7-point Likert scale (from “totally disagree” to “totally agree”) using a 3-item measure including “The information is hard to interpret/hard to extract/difficult to understand”. For objective understanding, an index was computed counting the number of correct answers to 14 multiple-choice questions on the definition and the interpretation of the star-rating information (scores from 0 to 14). To assess usage of the information, participants were asked to rate on a 7-point scale how often (ranging from “never” to “always”) they used the information (1) to make their meal choice, (2) to choose the healthiest meal, (3) to avoid meals containing too much energy, (4) to avoid meals containing too much saturated fat, (5) to avoid meals containing too much sodium (salt).

Two potential mediators derived from the HOE model were “effect on subjective knowledge” about the healthiness of a food and “effect on attitude” towards healthy canteen meals (Table 1). Subjective knowledge (4 items; Cronbach’s alpha: baseline = 0.75, follow-up = 0.81) [28] and attitude (single item) [29] were measured at baseline and follow-up by asking participants’ agreement with a series of questions on a 7-point Likert scale. Examples of items used to measure subjective knowledge are “I have a lot of knowledge about how to evaluate the nutritional value of a food”, “I have a lot of knowledge about how to prepare a healthy meal”, “I know which food is healthy for me” and “My friends consider me as an expert in healthy foods”. Attitude was measured using the following item “Canteen meals that are designated as healthy choices are better for me”. For the effect on subjective knowledge a score of one to five was assigned as follows: (1) negative change in knowledge, (2) maintaining low knowledge, (3) maintaining moderate knowledge, (4) maintaining high knowledge, (5) positive change in knowledge. The same classification procedure was used to derive effect on attitude with five final categories: (1) negative change in attitude, (2) maintaining low attitude, (3) maintaining moderate attitude, (4) maintaining high attitude, (5) positive change in attitude. A decrease and increase in knowledge or attitude from baseline to follow-up of more than one standard deviation (with SD = 0.94 for change in subjective knowledge; SD = 1.34 for effect on attitude) on a 7-point scale was used to classify participants under categories 1 and 5, respectively.

Both single and multiple items were used to measure mediator constructs. Multiple items are often preferred above a single item in order to capture more of the construct meaning and to enhance construct validity [30,31]. However, recent research has indicated that the predictive validity of single-item measures for concrete constructs are as predictive as multi-item measures [32]. Regarding the number of items to be considered, no concrete rule of thumb exists.

#### Theory-based moderators

The potential moderators of the intervention effects were defined on the basis of the objective nutrition knowledge and intention to change diet at baseline. Four subgroups of individuals were compared: those with (1) high knowledge and high intention (n = 44), (2) high knowledge and low intention (n = 54), (3) low knowledge and high intention (n = 70), (4) low knowledge and low intention (n = 52). Objective nutrition knowledge was determined using the index of knowledge on dietary recommendations developed by Grunert et al. [33]. High versus low knowledge was defined as a score of more versus less than 8.5 on 19 items. Participants’ intention to change their diet in the next six months (used as a proxy for motivation to change diet) was measured on a 7-point interval scale from “very unlikely” to “very likely” (5 items; Cronbach’s alpha = 0.94) by means of the following items: “I plan/expect/desire/intend/want to eat more healthy” [34]. A median split (cut-off = 4.7) was used to form high and low subgroups on intention of dietary change. The mean (SD) and the distribution of the moderators for the different subgroups are presented in Table 1.

### Statistical analyses

Data were analyzed using the robust maximum likelihood procedure in LISREL 8.72 [35]. First, a structural equation modelling (SEM) analysis was used to test the hypothesized model (Figure 1) for the total sample. Second, a multi-group SEM analysis was performed to investigate the same model in the four subgroups of individuals (characterized by moderating factors) and, as such, to provide insights in differences in the effectiveness of the intervention between the subgroups. SEM is a multivariate technique combining aspects of factor analysis and multiple regression that enables to simultaneously estimate a series of hypothesized relationships among observed and unobserved (latent) variables to determine whether these associations are consistent with an obtained sample of data [36,37]. The unique advantage of SEM is that by using latent variables, the measurement error can be eliminated. Extracting measurement error in SEM implies greater theoretical meaningfulness and cross-population stability to the parameters than might be achieved with methods such as regression or analysis of variance that do not correct for unreliability [38]. Additionally, SEM allows analysing simultaneously a system of equations that represent (full) theoretical models [36]. SEM also enables to examine relations between variables, such as mediators and moderators, in a simultaneous way (by means of multi-group analysis) that many other techniques cannot [37].

Correlation coefficients were first calculated between the variables of interest. All correlations were below 0.70, thus multicollinearity was not a concern in the present data [39]. SEM parameters were then estimated and the general fit of the model was assessed first for the total sample and then for the four subgroups based on nutrition knowledge and motivation to change diet. To evaluate the fit of the model, the *χ*^2^-value together with degrees of freedom are reported, as well as four other indices: the root mean square error of approximation (RMSEA), the normed fit index (NFI), the non-normed fit index (NNFI) and the comparative fit index (CFI). Values below 0.08 for RMSEA [40] and above 0.90 for NFI, NNFI and CFI [36] indicate an acceptable fit between the model and the data.

## Results and discussion

### Goodness-of-fit of the models

First in order to analyse the process by which the POP nutrition information achieved its effects on the meal choice and energy intake, a SEM was performed on the total sample. The hypothesized model as presented in Figure 1 performed well for the total sample (Table 2). The *χ*^2^ for the model was 172.58 with 74 degrees of freedom (p < 0.001). The RMSEA value was 0.078; the CFI was 0.96, the NNFI was 0.94 and the NFI was 0.93, indicating that the goodness-of-fit indices were satisfactory [36,40]. Second, in order to test whether the intervention was more effective in changing the meal choice of subgroups of university canteen customers, a multi-group analysis was performed. The data by subgroup fitted also the model well (but not as good as for the total sample). The *χ*^2^ for the model was 439.35 with 320 degrees of freedom (p < 0.001). The RMSEA value was 0.083; the CFI was 0.95, the NNFI was 0.93 and the NFI was 0.83.

**Table 2:** **Standardized solutions for hypothesized relationships between intervention, mediators and behavioural outcome for different groups**^**a**^

**Construct**	**Path**	**Construct**	**People with low knowledge and high intention (n = 70)**	**People with high knowledge and high intention (n = 44)**	**Total sample (n = 220)**^**b**^
Liking of information	$\overset{A}{\to }$	Use of information	0.58	0.90	0.59
Liking of information	$\overset{B}{\to }$	Effect on attitude	0.31		0.29
Use of information	$\overset{C}{\to }$	Effect on attitude			0.19
Objective understanding of information	$\overset{D}{\to }$	Use of information		0.38	
Use of information	$\overset{E}{\to }$	Effect on energy intake		0.46	
Subjective understanding of information	$\overset{F}{\to }$	Use of information		−0.27	
Objective understanding of information	$\overset{G}{\to }$	Subjective understanding of information	0.28		0.20
Subjective understanding of information	$\overset{H}{\to }$	Effect on energy intake			−0.18
Subjective understanding of information	$\overset{I}{\to }$	Effect on subjective knowledge			0.17
Use of information	$\overset{J}{\to }$	Effect on subjective knowledge			0.24
Effect on attitude	$\overset{K}{\to }$	Effect on energy intake		0.41	

^a^Only paths with at least one significant coefficient in any of the three models are included.

^b^Four individuals were removed from the sample because of incomplete information, leaving a final sample of 220 valid cases.

### Role of liking

For the total sample and each of the four investigated subgroups, a significant relation between liking and use of the information was observed (Path A in Table 2). This association was also found to be the strongest, indicating that people who liked the information more, declared to use the information more often. A moderate significant path from liking of the information to a positive effect on attitude or maintenance of high attitudes was observed in the total sample, both directly (Path B) and indirectly (Path C) through claimed usage of the information. The direct path from liking to effect on attitude was also found in the subgroup with low knowledge and high motivation (Path B).

Compared to the understanding of the information, liking was a more important predictor of information use. This finding highlights the need for communication efforts and research to move beyond a focus on “understanding of nutrition information” and to emphasize more the liking and attractiveness of information formats. It seems that most consumers have a reasonable understanding of nutrition information when prompted, but only a minority seems to look for nutrition information when shopping [33]. The present study confirmed the general good level of objective understanding of POP nutrition information with less than 10% of the sample having a score of below 7 on 14. Liking of the information was more heterogeneous among the sample with 25% having a score of less than 4 on a 7-point scale. These findings suggest that information characteristics (e.g. display size, colour scheme), which are key determinants of consumers’ attention to nutrition information [41] and liking of the information [42], may offer a window of opportunity to improve the effectiveness of nutrition information in terms of targeted dietary change.

### Nutrition knowledge versus motivation

Participants needed both to like and (objectively) understand the information to use it (Path A; Path D), leading to a decrease in energy intake or maintenance of the recommended energy intake level (Path E), as shown in the subgroup of high motivated and knowledgeable consumers. Objective knowledge has also previously been reported to act as a moderator of the relation between objective understanding and use of the information on one hand [33], and between use and effect on energy intake on the other hand [15]. Compared to this subgroup, the remainder of the total sample reported a significantly lower objective nutrition knowledge (p < 0.001). Although simplified nutrition information does not require detailed nutrition knowledge [25]; some level of knowledge seems necessary to result in effective usage of the information [12]. Moreover, higher nutrition knowledge may also indicate a higher interest in nutrition and healthy eating [43]. These findings suggest the need for more nutrition education.

A more important moderator of participants’ responses to nutrition information was their motivation to change diet as illustrated by the outcome of the multi-group analysis, which was also consistent with previous studies [44,45]. In addition to nutrition education, the challenge is to investigate how to motivate people (more) to change dietary habits.

### Objective versus subjective understanding of the posted information

The distinction between objective and subjective understanding was first made by Grunert & Wills [23], but no study thus far analyzed the importance of subjective understanding in explaining consumers’ use of nutrition information. In this study, no significant association was found between subjective understanding and the use of the information, except for the subgroup with high knowledge and high motivation, for whom the relation was negative (Path F). A possible explanation was that the more knowledgeable participants were, the more they tended to underestimate their own performance compared with that of peers [46]. In the total sample and in the subgroup of participants with low knowledge and high motivation, a significant association between objective and subjective understanding was observed (Path G).

Moreover, for the total sample, subjective understanding was negatively associated with an effect on energy intake (Path H), but positively with an effect on subjective knowledge (Path I). This could indicate that an important segment of our sample was in a learning stage – hence, not (yet) ready for action – which is comparable to the motivational phase as defined by Renner and Schwarzer [47] and the contemplation or preparation stage of change described by Prochaska & Velicer [48]. Our results suggest this learning path, but formal confirmation needs further investigation. Simultaneously, the same relations between subjective understanding and effect on energy intake on one hand and subjective knowledge on the other hand were observed in the subgroups with low motivation (results not shown), which indicated that part of this learning segment may probably never evolve to behavioural change because of a lack of personal motivation. Again the importance of personal motivation is highlighted.

### Intervention effect on subjective knowledge

An increase in subjective knowledge or maintenance of high subjective knowledge was found in the total sample to result from a higher use of the information (Path J), but not because of a higher objective understanding of the information. Subjective knowledge is usually defined as people’s subjective perceptions of what or how much they know about a specific product compared with peers [49,50]. In the present study, we did not measure the perceived knowledge of products but of skills (i.e. to evaluate the nutritional value and healthfulness of a food), which is often referred to as self-efficacy [51]. Previous studies have indicated that although nutrition information may not have an immediate effect on food choices and dietary intake, such information may act together with other factors to enhance consumers’ self-efficacy and thereby increase the likelihood of healthier food choices being made later on [10,52]. Therefore, a nutrition-information intervention that targets self-efficacy may, in the long run, lead to dietary changes.

### Relation between attitude and behaviour

The results for the subgroup of high motivated and knowledgeable consumers support a positive relation between attitude towards healthy eating and dietary behaviour (Path K) [53,54]. Again this suggests that some baseline level of nutrition knowledge may be necessary to translate a positive attitude into a lower energy intake. In general, the attitude towards healthy canteen meals decreased after posting the information (paired sample’s *t*-test p < 0.001). In both the baseline and follow-up periods healthy meal options or three-star meals constituted a minority of the overall meal offer at the canteens (i.e. about 5%). A previous study showed that most consumers seem to underestimate the nutritional quality of foods when eating out [6]. Therefore, a possible explanation for the negative change in attitude is the increase in canteen customers’ awareness of the actual healthiness of the meals offered after posting the nutrition information. Improving this attitude by increasing the offer of healthy choices might therefore be an important step forward in the development of effective strategies for stimulating healthier meal choices.

### Strengths and limitations of the study

The major strength was the application of a new advanced approach to the evaluation of intervention effects in nutrition research. Another strength of the study was the careful follow-up of the daily food consumption of young adults and its determinants in a real-life setting. Some limitations should also be acknowledged. First, the use of a convenience sample limits the interpretation of the findings to its specific sampling frame. Extrapolation to other populations remains to be further validated. Second, the small sample size did not allow to use the midpoint of 4 to discriminate between participants with a high versus low intention to change their diet. Moreover, the limited sample size may have reduced the ability to detect significant differences in more personal factors with sufficient power. Third, the limited duration of follow-up did not permit evaluations of gradual behavioural changes and persistence of behavioural change over time. Fourth, regarding the hypothetical model, other sequences of hierarchical stages than one from cognitive processing to a behavioural outcome, might have been valuable as well to explain the (in)effectiveness of the nutrition-information intervention. Therefore, further investigation of alternative sequences of hierarchical effects is recommended. A final limitation pertains to the choice of dependent variable where the energy intake from the canteen meal was used as a proxy for the star rating of the reported meal choice, which implies that the analyses do not directly map onto the failed intervention. For example, an individual may have intentions to increase his/her vegetable intake as a result of the intervention but this may not necessarily affect his/her energy intake from the meal. However, from all targeted nutrients/food group in the intervention (i.e. energy, saturated fat, salt and vegetable portion), the energy content had the highest discriminative power for the healthfulness of a meal choice [9]. The main argument for not using meal choice as outcome measure, is that in that case the variance in the dependent variable would have been substantially reduced due to the conversion of an ordinal variable (i.e. star rating of a meal) into a categorical variable, resulting in a considerable loss of information.

## Conclusions

The proposed moderated mediation model of nutrition-information effects contributed to a better understanding of the ineffectiveness of a nutrition-information intervention in university canteens. The model highlighted the importance of liking of the posted information. The nutrition information was more effective for the more motivated students and for those with a greater objective nutrition knowledge. Increasing students’ motivation to change their diet and, to a lesser extent, their knowledge is recommended. Additionally, creating an eating environment with more healthy choices and attractive POP nutrition information complemented with the provision of nutrition education, is proposed for the development and implementation of effective nutrition-information strategies.

## Abbreviations

HOE: Hierarchy-of-effects; POP: Point-of-purchase; SD: Standard deviation; SEM: Structural equation modelling; RMSEA: Root mean square error of approximation; NFI: Normed fit index; NNFI: Non-normed fit index; CFI: Comparative fit index.

## Competing interests

The authors declare that they have no competing interests.

## Authors’ contributions

CH conducted the data collection and processing. Furthermore, CH interpreted the data and drafted the manuscript. ZP performed the statistical analyses and participated in the data interpretation and drafting of the manuscript. WV and JVC revised the manuscript for important intellectual content. All authors contributed to the conceptualization and design of the study. All authors read and approved the final manuscript.
